# Comparison of different registration methods and landmarks for image-guided radiation therapy of pulmonary tumors

**DOI:** 10.1186/s12880-019-0343-3

**Published:** 2019-05-31

**Authors:** Xiaohui Cao, Ming Liu, Fushan Zhai, Nan Li, Chaoen Bao, Yinliang Liu, Gang Chen

**Affiliations:** 1 Department of Radiotherapy and Oncology, The Third Hospital of Hebei Medical University, Shijiazhuang, 050051 Hebei China; 2 Department of Respiratory Medicine, The Third Hospital of Hebei Medical University, 139 Ziqiang Road, Shijiazhuang, 050051 Hebei China

**Keywords:** Cone beam computed tomography, Image-guided, Image registration, Lung cancer

## Abstract

**Background:**

To compare the accuracy, advantages and disadvantages of automatic registration methods at different anatomical-sites for thoracic image-guided radiation therapy (IGRT).

**Methods:**

The Varian-IX IGRT system was used to perform a manual registration of the images collected on the first fraction of 60 patients with lung cancer (42 cases central location and 18 cases of peripheral). The registered images were used as reference images. Offline registration was performed for computed tomography-CBCT images using four methods: whole image registration, ipsilateral registration, soft tissue tumor registration, and vertebral body registration. Time taken to complete and deviation value were analyzed between the different methods.

**Results:**

There were significant differences in absolute deviation value of all the three directions (*P* < 0.001) and the time consumption (*P* < 0.001) between 4 methods. The Z direction had significant differences in deviation value of 4 methods (0.023 ± 0.128 mm, − 0.030 ± 0.175 mm, − 0.010 ± 0.238 mm, − 0.075 ± 0.137 mm, *P* = 0.011). The difference was significant in the X direction of the ipsilateral registration method between central and peripheral lung cancer (0.033 ± 0.053 mm vs. 0.067 ± 0.067 mm, *P* = 0.045).

**Conclusions:**

The whole lung or affected side registration methods could be recommended to be used in the automatic registration function of the Varian-IX’s On-Board Imaging (OBI) system.

## Background

The morbidity and mortality of lung cancer rank first among all malignancies in China [1–3]. Radiation therapy (RT) is an important treatment modality for lung cancer [4, 5]. Requirements of precision on the treatment site are higher with the increasing application of intensity-modulated radiation therapy (IMRT) [6, 7]. Image-guided radiation therapy (IGRT) provides the basis for the precise treatment of IMRT [8, 9]. Cone-beam computed tomography (CBCT) scans have widely accepted clinically in confirming daily patient positioning [10].

IGRT is used to validate treatment position before administering treatment, to measure and analyze the three-dimensional deviation value at the central sites of tumors, as well as perform correction, which is currently one of the most important bases of precise treatment [8, 9]. CBCT is applied widely in IGRT systems, but CBCT involves a cone-beam scan, which not only has less resolution than fan-beam CT, but also takes longer time to acquire the images [11]. In addition, lung tumor imaging requires several breathing cycles, increasing scanning time and patient inconvenience [12]. Therefore, developing a standard to rapidly and accurately register CBCT planning images is of clinical significance in this subset of patients.

A large number of studies explored CBCT in position verification [13–15], but few studies are available on the accuracy of automatic registration of on-board imaging (OBI) systems as IGRT imaging system. In this study, CBCT images and planning CT images of 60 patients with lung cancer were registered. Different registration methods and registration regions were applied for tumors in different lung areas. Moreover, registration accuracy, as well as pros and cons of different automatic registration conditions were compared to provide a reference for clinical registration.

## Methods

### Study design

This study was a retrospective study of 60 patients with lung cancer treated at the Third Hospital of Hebei Medical University (China) between March 2012 and December 2013.The study was approved by the ethics committee of the Third Hospital of Hebei Medical University. The need for individual consent was waived by the committee.

The inclusion criteria were: 1) pathological diagnosis of lung malignancies; 2) underwent radiotherapy; 3) underwent CBCT scan at least once every week; and 4) patients aged 18–85 years.

Patients with an inadequate number of scans or patients with poor-quality images were excluded from this study.

### CT scan

The patients were simulated in the supine position. A pillow was placed under the patients head to keep them in a comfortable position. The patients’ hands were placed on their forehead and were fixed in position with a thermoplastic film. The patients breathed calmly, and thoracic CT scan was performed using a Somatom-sensation Plus-16 spiral CT scanner (Siemens, Erlangen, Germany) with a 5 mm slice thickness. The scanning range was from the cricothyroid membrane to the lower edge of the diaphragm. The scanned images were transmitted to the CMS treatment planning system (CMS, Inc., St Louis, MO, USA).

### Acquisition of CBCT images

CBCT scan and treatment were performed using an IXOBI linear accelerator (Varian Medical Systems Inc., Palo Alto, CA, USA). First, a conventional fractionation was prescribed and CBCT scan was conducted for all patients. CBCT scans were then performed one to two times every week on treatment. CBCT scan was performed before each treatment for patients who underwent hypo-fractionation. An offline study was performed using CBCT images scanned on the first fraction for each patient.

### Comparison of the settings of OBI automatic registration regions and registration methods

Many authors believe that gray-scale registration is more accurate than other types of registration [16]. Therefore, in this study, we used gray-scale registration. CBCT images for each patient and positioning images were registered manually to reduce the effects of placement deviation value on the registration results. The optimal position was confirmed by two specialists in imaging, the deputy chief physician and a researcher in the imaging department, which was then used as the reference standard. The reference criteria for manual registration were the comprehensive evaluation that the tumor, spine, pulmonary tissues, and the overall body were in the best status. The criteria for manual matching were as follow. The tumor and normal tissues had to be perfectly matched after manual matching. The matching of the tissues including tumor, spinal cord, lung tissues, and heart had to be confirmed in this order. In order to avoid errors, the matching had to be confirmed independently by the physician in charge of the patients and the investigators. Perfect matching could be achieved in some patients, but the matching of the tumor and spinal cord were not perfect in some patients, due to various causes. For such patients, according to the requirements of the treatments in clinical practice, if the tumor were distant from the spinal cord (the radiation dose to the spinal cord was low), matching of the tumor was the priority. For patients in whom the tumor was close to the spinal cord (the radiation dose to the spinal cord would exceed the maximum tolerated dose), matching of the spinal cord was the priority. Generally, the tumors are well visualized in the patients receiving radiotherapy, but for some patients with relatively small tumor or specific tumor sites, the display of the tumor was actually not as clear as using positioning CT. In these cases, the window level and window width were adjusted, and the largest layer of the tumor center was selected, in order to compare the matching. Deviation values in X, Y, and Z directions were set to 0. Then, offline registration was performed for CT-CBCT imaging using four registration methods: whole registration (all slices in the range of CBCT scanning), registration of the ipsilateral side (tissues of the affected side in CBCT scanning range), tumor registration (margin of 1 cm in gross tumor volume three-dimensional directions) and vertebra registration (the whole vertebral body in the CBCT scanning range in three-dimensional directions), respectively. Registration was performed using OBI Offline Review 8.6 (Varian Medical Systems Inc., Palo Alto, CA, USA).The deviation value of the target centers of the scanned images and the planning images in the left-right (LR), superior-inferior (SI), and anterior-posterior (AP) directions were recorded. The differences in time consumption and accuracy of different registration methods were also analyzed.

### Statistical analysis

A one-way analysis of variance was performed for the registration results in different regions. The least significant difference was used for pair-wise comparison. Statistical analysis was performed using SPSS13.0 (SPSS Inc., Chicago, IL, USA) software. A *P* value < 0.05 was considered to be statistically significant.

## Results

### General clinical data

Sixty patients with thoracic malignancies were included in this study. This included 35 cases of left lung cancer and 25 cases of right lung, 42 cases of central lung and 18 cases were peripheral lung cancer. All patients were pathologically confirmed (Table 1).

**Table 1:** General clinical data

Characteristic	Value
Targets	65
Patients	60
Age, median (range) (years)	67 (40–84)
Gender, *n* (%)	35 (58)
Weight, median (range)	67 (48–92)
Primary NSCLC, *n* (%)	56 (93)
Pulmonary metastasis, *n* (%)	4 (7)
Tumor size	
Diameter of tumor (cm), mean ± SD	5.9 ± 2.2
Range (cm)	1.5–12
Target number of each patient	
One target	56
Two targets	3
Three targets	1
Tumor locations	
Left	35
Right	25
Central type	42
Peripheral type	18

### Comparison between the four registration methods

A total of 240 groups of registration data were available for the 60 patients. The deviation values of the image registration methods in the X, Y, and Z directions (LR, SI, and AP directions) are shown in Table 2. The values shown are from automatic registration in the four registration regions after manual registration. This was to reduce the effects of placement deviation value on the registration results. Therefore, the deviation values were small. The analysis revealed statistically significant differences in the Z direction, and the differences in the time consumed were large between the four methods (Table 2).

**Table 2:** Deviation value, time consumed and statistical values at different registration sites in X, Y, and Z directions (x ± s)

Registration sites	Whole registration	Ipsilateral side registration	Tumor registration	Vertebra registration	*P*
Time consumed (s)	18.9 ± 4.3	4.9 ± 0.9	2.2 ± 0.4	3.1 ± 0.2	< 0.001
Absolute value					
X direction (mm)	0.042 ± 0.056	0.043 ± 0.059	0.098 ± 0.091	0.113 ± 0.096	< 0.001
Y direction (mm)	0.108 ± .129	0.135 ± 0.138	0.148 ± 0.155	0.233 ± 0.206	< 0.001
Z direction (mm)	0.077 ± 0.105	0.123 ± 0.128	0.170 ± 0.166	0.118 ± 0.102	< 0.001
Deviation value					
X direction (mm)	0.002 ± 0.070	0.010 ± 0.073	0.018 ± 0.133	0.023 ± 0.148	0.646
Y direction (mm)	−0.012 ± 0.169	−0.018 ± 0.193	− 0.028 ± 0.213	− 0.047 ± 0.309	0.770
Z direction (mm)	0.023 ± 0.128	− 0.030 ± 0.175	− 0.010 ± 0.238	− 0.075 ± 0.137	0.011

DICOM standard

Deviation values were directional, including positive and negative deviation values. However, the absolute deviation value is more important in clinical procedures. If the directionality of the deviation value was not taken into consideration, the absolute deviation value in the (LR, SI, and AP directions) all showed statistically significant differences between methods (Table 2).

Stratified analysis was performed for central and peripheral types, which showed that the differences in L-R direction of affected side was significant (*P* = 0.045, shown in Table 3).

**Table 3:** Comparison of patients with central and peripheral lung cancers in X, Y, and Z axes (*P* value)

	Central type (*n* = 42)	Peripheral type (*n* = 18)	*P*
Age (median, range)	67.0 ± 8.9	67.4 ± 10.4	0.869
Gender (*n*, %)	25, 60%	10, 56%	
Weight	65.6 ± 10.0	72.3 ± 11.4	0.027
Primary NSCLC	42	14	0.002
Pulmonary metastasis	0	4	
Tumor size			
Diameter of tumor (cm) (mean ± SD)	6.2 ± 2.2	5.2 ± 2.2	0.095
Range (cm)	1.67–12.00	1.5–9.00	
Target number of each patient			0.007
One target	42	14	
Two targets	0	3	
Three targets	0	1	
The whole lung (absolute value)			
X direction (mm)	0.043 ± 0.059	0.039 ± 0.050	0.804
Y direction (mm)	0.093 ± 0.116	0.144 ± 0.154	0.158
Z direction (mm)	0.081 ± 0.117	0.067 ± 0.069	0.632
Affected side (absolute value)			
X direction (mm)	0.033 ± 0.053	0.067 ± 0.067	0.045
Y direction (mm)	0.136 ± 0.139	0.133 ± 0.137	0.952
Z direction (mm)	0.110 ± 0.103	0.156 ± 0.172	0.205
Tumor (absolute value)			
X direction (mm)	0.086 ± 0.075	0.128 ± 0.118	0.102
Y direction (mm)	0.148 ± 0.157	0.150 ± 0.154	0.957
Z direction (mm)	0.162 ± 0.132	0.189 ± 0.230	0.568
Vertebral body (absolute value)			
X direction (mm)	0.102 ± 0.078	0.139 ± 0.129	0.181
Y direction (mm)	0.219 ± 0.203	0.267 ± 0.214	0.416
Z direction (mm)	0.107 ± 0.095	0.144 ± 0.115	0.195

## Discussion

Manual image registration using the tumor as the marker after a CBCT is often used in the clinical setting. [17]. If a significant difference exists in the density between tumor and the surrounding pulmonary tissues, the tumor itself has the basic conditions as a registration marker [18]. The obtained CBCT images contain information of respiratory motion because the acquisition of these images takes a long time and the tumors move with breathing [19], which can affect the accuracy of image registration. Furthermore, tumors may become smaller with treatment, leading to a significant difference in planning images and difficulties in image registration. Therefore, anatomical structures that have a significant difference in density compared with the surrounding tissues and no significant changes with treatment are considered as more reliable marker, such as vertebral body and carinas. Higgins et al. [20] showed that the spine and carina can be used equally for CBCT image registration without compromise, but the carina could be more reproducible than the spine. Castillo et al. [21] showed that the use of landmark pairs for deformable image registration led to a narrow uncertainty range. For 4D CBCT, Li et al. [22] showed that the use of spine, spine plus internal target volume, and lung resulted in similar results. The registration quality of CBCT scanning images and planning CT images plays a decisive role in accurately evaluating the placement deviation value. Therefore, Ottosson et al. [23] proposed that the placement deviation values obtained by different registration methods were different, and different margins were needed. Therefore, this study focused on whether different registration regions would affect registration results after excluding the effects caused by placement deviation value. Manual registration implies that CBCT images are manually adjusted to be exactly the same as planning CT images, which is considered to be the best registration method. This can be a lengthy procedure, affecting work flow efficiency. Automatic registration methods take less time; integrate data only from the region of interest, and different registration regions may have different registration results. In this study, images manually registered were used as the reference images, and automatic registration results of different anatomical regions were compared to evaluate differences. The findings demonstrated no statistically significant differences in X and Y directions between the four methods compared with the reference method. The pair-wise comparison found that the vertebral body method was statistically different from the total registration, tumor, and reference methods, but no statistically significant difference was found in the other groups. The reason being was that positioning CT images were instant images, while CBCT images were a fusion of phases in several respiratory cycles due to long scanning time. Breathing had a certain impact on the expansion of pulmonary tissues, and tumor size and location, but it had relatively less impact on the vertebral body [12]. The impact of the respiratory cycle was considered in the cases of whole registration, pulmonary tissue registration, tumor registration, and manual registration were used, but it was not considered in the case of vertebra registration. A statistically significant difference was found in the Z direction as it was less affected by other factors. This should be more meaningful to provide reference for clinical application.

Lung cancer can be categorized as both central lung cancer and peripheral lung cancer according to the relationship between the diseased region and the bronchus. A lung tumor can move in all three-dimensional directions, and the closer to the diaphragm the tumor is located, the greater the chance of movement. The impact of respiratory movement on central lung cancer was different compared with that on peripheral lung cancer. Therefore, some studies suggested that the anatomical registration markers were different between central and peripheral lung cancers [24]. In this study, a stratified analysis was performed for absolute deviation value of central and peripheral lung cancers, comparison between methods was not statistically significant in Y and Z directions, with the differences in X direction of affected side were being found significant.

The time consumed for image registration was approximately 19 s, for the whole registration, 2 s affected side, 3 s for the tumor registration and 5 s for vertebra registration methods, all with significant differences observed. It was found, the larger the selected registration range, the more the integrated data and the longer the time taken. If the directionality of deviation value was not taken into consideration, and only value differences were compared, it could be concluded that significant differences exist in different registration regions. Clinically, this study focused on the deviation value of the actual target center and the target center of planning images in the LR, SI, and AP directions, which were geometric deviations.

Tumors may respond and become smaller during the treatment process, and some tumors may not be significantly different in density compared with the surrounding tissues, leading to difficulty in registration. Vertebra registration was significantly different from the reference images and other registration methods. Although this study demonstrated that the error was small, the results excluded placement deviation value. If the placement deviation value was considered, the difference would be greater. The accuracy of integrated registration satisfied the clinical requirement, but increased time, thus increasing the waiting time of the patients during treatment. A comparison of the four registration methods indicated that it could be recommended to use gray-scale automatic registration with the affected side as the registration region.

In practical treatment, the anatomical structures in the planning systems and those in the practical treatment are different due to various reasons, this can include iso-center consistency among CT simulators, planning systems, and treatment machines, changes in body structures caused by daily positioning, movement during treatment, change in weight, filling and movement of organs in the target region or adjacent region; impact of normal respiratory movement and heartbeat, and changes in volume and location of tumors as well as surrounding normal organs due to radiotherapy dose and chemicals. Therefore, automatic registration may have its own advantages and disadvantages, but in practice, clinicians are still recommended to perform manual adjustments based on anatomical markers after automatic registration to achieve the most clinically acceptable image registration.

### Limitations

This study had certain limitations. Firstly, the sample size collected was small and from a single center. Therefore, a larger-sample size is required to achieve better results. Secondly, the study had no gold standard to estimate the deviation or absolute value owing to the lack of consensus in the current guidelines and literature. Finally, matching breathing-free CT with CBCT is a limitation of the study. For the institutions already using 4D-CT, matching by the mean density would be ideal, but due to the specific requirements of the 4D-CT scanner, as well as the specific conditions of some patients (such as irregular or uneven respiration, chest distress, shortness of breath, and poor cooperation), 4D-CT is still not widely used in clinical practices yet. Thus this study aimed to provide some evidence using the facilities and data available at our hospital. Additional studies are still necessary to determine the best registration methods for specific contexts. Comparisons of different systems could also be performed.

## Conclusion

In conclusion, the whole lung or affected side registration methods are be recommended to be used in the automatic registration function of the Varian-IX’s OBI system, with the use of vertebral registration might not be advisable after comprehensive consideration of the collected data.

## Data Availability

The datasets used and/or analyzed during the current study are available from the corresponding author on reasonable request.

## Abbreviations

AP: Anterior-posterior
CBCT: Cone-beam computed tomography
IGRT: Image-guided radiation therapy
IMRT: Intensity-modulated radiation therapy
LR: Left-right
OBI: On-board imaging
RT: Radiation therapy
SI: Superior-inferior
